# Migraine Pharmacological Treatment and Cognitive Impairment: Risks and Benefits

**DOI:** 10.3390/ijms231911418

**Published:** 2022-09-27

**Authors:** Mirella Russo, Matteo A. De Rosa, Dario Calisi, Stefano Consoli, Giacomo Evangelista, Fedele Dono, Matteo Santilli, Alberto Granzotto, Marco Onofrj, Stefano L. Sensi

**Affiliations:** 1 Department of Neurosciences, Imaging and Clinical Sciences, “G. d’Annunzio” University of Chieti-Pescara, 66100 Chieti, Italy; 2 CAST—Center for Advanced Studies and Technology, “G. d’Annunzio” University of Chieti-Pescara, 66100 Chieti, Italy; 3 Institute for Mind Impairments and Neurological Disorders-iMIND, University of California, Irvine, Irvine, CA 92697, USA; 4 ITAB—Institute of Advanced Biomedical Technology, “G. d’Annunzio” University of Chieti-Pescara, 66100 Chieti, Italy

**Keywords:** migraine, dementia, Alzheimer’s disease, neurodegeneration, neuroprotection

## Abstract

Migraine is a common neurological disorder impairing the quality of life of patients. The condition requires, as an acute or prophylactic line of intervention, the frequent use of drugs acting on the central nervous system (CNS). The long-term impact of these medications on cognition and neurodegeneration has never been consistently assessed. The paper reviews pharmacological migraine treatments and discusses their biological and clinical effects on the CNS. The different anti-migraine drugs show distinct profiles concerning neurodegeneration and the risk of cognitive deficits. These features should be carefully evaluated when prescribing a pharmacological treatment as many migraineurs are of scholar or working age and their performances may be affected by drug misuse. Thus, a reconsideration of therapy guidelines is warranted. Furthermore, since conflicting results have emerged in the relationship between migraine and dementia, future studies must consider present and past pharmacological regimens as potential confounding factors.

## 1. Introduction

Migraine is the most prevalent non-communicable neurological disorder in subjects in the 35–60 age bracket, whereas dementia leads the ranking in older individuals [1]. The two conditions are significantly debilitating and represent a significant burden on health systems [1]. Recent investigations have attempted to assess links between the two conditions, but, in most cases, the results were conflicting and inconclusive [2]. While an increased risk for mid-to-late-life cognitive impairment in migraineurs [3,4,5,6,7] has been frequently reported, fewer observations have contradicted the notion [8,9]. Longitudinal evaluations of the cognitive status of people with migraine (PwM) have shown average performances [10,11]. In these surveys, the risk of dementia has also been evaluated according to patients’ features and subtypes, with mixed results. For instance, the increased risk of dementia has been reported only in female subjects by some authors [5,6], but others also indicated the opposite [3,12]. Finally, a recent meta-analysis confirmed the existence of a minor yet significant increased risk for all-cause dementia (RR = 1.33; 95%CI: 1.16–1.5) [13].

An unresolved issue concerns the type of dementia associated with cognitive impairment. 

Alzheimer’s disease (AD) is the most common cause of dementia in the elderly [14]. Thus, the present review will be focused on this pathology. The current understanding of AD pathophysiology is highly debated. Nevertheless, the disease’s pillars are represented by the accumulation of brain deposits of β-amyloid and neurofibrillary tangles of hyperphosphorylated tau [15,16]. Other dementias include Lewy body dementia (LBD), which encompasses dementia with Lewy bodies (DLB) and Parkinson’s disease with dementia (PDD), frontotemporal dementia (FTD) [17], and limbic-predominant age-related TDP-43 encephalopathy (LATE) [18]. 

Most migraine therapies last several months or years and directly affect the central nervous system (CNS) [19,20]. Migraine treatment aims to interrupt painful attacks with abortive drugs and, when needed, prevent new attacks with prophylactic interventions. Nonsteroidal anti-inflammatory drugs (NSAIDs), such as naproxen, aspirin, diclofenac potassium, celecoxib, and ibuprofen, are the first-line treatment for migraine attacks [19,20]. Combinations of drugs, such as acetaminophen + aspirin + caffeine, are also employed, as well as specific second-line or third-line migraine treatments, such as triptans (II), ditans (III), and gepants (III) [20]. Ergot-derivates are less frequently used (see Section 3.8) [20,21]. Prescription of a preventive treatment has been recommended for ≥4 days of migraine-related disability per month [21]. However, new guidelines indicate that it should be employed in the case of ≥2 days of impairment despite optimized first-line therapy [20]. Albeit not mandatory, this indication may lead to widespread and long-term use of compounds that affect the CNS. Migraine prophylaxis is recommended for at least three months but may be used up to 6–12 months [22]. Beta-blockers, topiramate, and candesartan are first-line medications. Flunarizine, amitriptyline, and sodium valproate are second-line, and GRP monoclonal antibodies are third-line treatments [20]. 

The present work reviews the mechanisms of action of pharmacological treatments for migraine, including abortive and prophylactic drugs. The goal is also to assess pharmacodynamic links with cognitive symptoms or AD-related neurodegenerative pathways. 

## 2. Cognitive Profiles in Migraineurs

### 2.1. Overview

Detailed descriptions of the subclinical neuropsychological features of PwM are beyond the scope of the present paper, and comprehensive reviews are already available on the subject [23,24,25]. Thus, only the main findings will be reported in this section. Mild visual and verbal memory impairment, reduced information processing speed, executive dysfunction, and attention deficits have been reported in PwM [23]. In the ictal phase, migraineurs may experience cognitive inefficiency and confusion that may be partially due to the concurrent physical symptoms, such as pain, nausea, and photophobia [26,27]. However, migraine is associated with slight cognitive dysfunctions also during the interictal periods. These appear in tasks involving complex attention and executive functioning, including set-shifting, spatial cognition, and immediate and delayed memory [24,25,28]. The deficits also affected global cognition, with a more prominent effect in PwM without aura [24]. They are influenced by the frequency and severity of the attacks [29]. Interestingly, the language domain is spared, whereas conflicting results are available on basic attention [24,25]. Of note, a negative correlation between mood and cognitive performance has been posited [25], possibly underlying the presence of shared network derangement. The overlap also creates a therapeutical window as some antidepressants are used as a migraine prophylaxis.

### 2.2. Small-Vessel Disease, Vascular Dementia, and Genetic Conditions

A striking intersection point between cognitive impairment and migraine is represented by vascular dementia (VaD). Increased cerebrovascular risk is acknowledged in migraineurs, whose brains typically display white matter changes and may also have small infarct-like lesions, especially in migraine with aura [30]. While most brain lesions are silent, a two-fold risk of ischemic attack has also been observed [31]. The ischemic strokes may be temporally related to the migraine attacks (migrainous infarction) [32] or can also be disjoined. Accordingly, cognitive impairment is evident immediately after acute brain infarction. On the other hand, a link between migraine and long-term development of VaD (e.g., due to chronic small vessel disease) was rarely confirmed [33] and challenged mainly by subsequent investigations [5,12,34]. 

Different from sporadic cases, some genetic-driven disorders show major overlap between migraine and vascular cognitive impairment. Cerebral autosomal dominant arteriopathy with subcortical infarcts and leukoencephalopathy (CADASIL), as well as mitochondrial encephalopathy, lactic acidosis, and stroke-like episodes (MELAS), are featured by migraine attacks and cognitive impairment, mostly related to progressive impairment in the cerebral white matter due to vascular damage [35,36]. Another genetic condition that encompasses cognitive impairment—mostly due to vascular damage and brain atrophy—and migraine is represented by retinal vasculopathy with cerebral leukoencephalopathy (RVCL) [37]. 

Cerebral amyloid angiopathy (CAA) is a neurodegenerative disorder, sharing pathophysiological and clinical traits with AD, that also combines cognitive impairment and migraine, to the point that migraine with aura attacks was proposed as an early biomarker of the clinical onset of a genetic variant of CAA (Dutch type) [38]. Of note, migraine attacks are also present in presenilin-1- and amyloid-precursor-protein-related familial AD [39]. 

## 3. Migraine Drugs, Neurodegenerative Pathways, and Cognition: Protection or Risk?

### 3.1. Anti-Seizure Medications (ASMs)

Data regarding cognitive adverse effects (CAEs) of ASMs have mainly come from studies performed on epileptic patients. Though some of these effects are undoubtedly the result of chronic exposure, CAEs onset can also be observed in medium-term administration as performed in migraine prophylaxis. 

Topiramate (TPM), valproic acid (VPA), and gabapentin (GBP) are currently the most frequently used ASMs in patients with chronic migraine as prophylaxis treatment [40]. 

**TPM** exerts its anti-migraine activity by stimulating GABA-A receptors, inhibiting AMPA and kainate receptors, and blocking cortical spreading depression (CSD), a neurophysiological phenomenon characterized by abrupt changes in intracellular ion gradients and sustained depolarization of neurons [41,42]. In preclinical models, inhibition of AMPA receptors has been linked to modulation of the Akt/GSK-3β/CREB neuroprotective pathway [43] and decreased oxidative stress and release of inflammatory mediators [44]. TPM also promotes neural homeostasis by stimulating the release of brain-derived neurotrophic factor (BDNF) [44]. CAEs are not uncommon with TPM and usually occur, in a dose-dependent fashion, during the early phase of the treatment (i.e., within the first 6 weeks). These side effects include attention deficits, psychomotor slowing, language and comprehension difficulties, short-term memory and working memory deficits, poor verbal fluency, reduced cognitive speed, and altered thinking [45,46,47,48,49,50]. About 10% of adult patients treated with TPM as prophylaxis for migraine attacks show mild-to-moderate concentration and memory difficulties, especially during titration [51,52]. These effects are reversible and generally disappear after treatment interruption. TPM has been shown to exert more negative effects on cognition than a range of other antiepileptic drugs [49]. Patients with pre-existing cognitive dysfunction or a past psychiatric history seem more prone to develop TPM-related CAEs [53,54]. The mechanism through which TPM may define the onset of CAEs is unclear. Experimental models suggest that TPM, like other sulfamate compounds (e.g., zonisamide, ZNS), exhibits a particular tropism for frontal areas, explaining the worsening in verbal fluency [55].

**VPA** is a voltage-gated sodium, potassium, and calcium channels blocker, which also increases GABA levels by inhibiting GABA aminotransferase [56]. VPA exerts its anti-migraine effects by blocking CSD. VPA administration generates only minor changes in cognitive functioning regarding attention and visuomotor performances [57]. A randomized controlled study in 480 treatment-naïve adult patients with focal epilepsy showed worse scores in attention and visuomotor tasks in the VPA-treated group compared to controls [58]. An additional study demonstrated that only a minor percentage of VPA-treated patients with epilepsy (1.3%) developed Parkinsonism, psychomotor slowing, and memory difficulties, with a delay of 2.5–10 months after treatment start [59]. These effects were unrelated to VPA dose, patient age, or epilepsy duration. On the other hand, acute and chronic VPA administration increase brain levels of taurine and glycine, as well as serotonin and dopamine in the hippocampus [60], improving cognitive functioning. In addition, VPA reduced β-amyloid generation and tau hyperphosphorylation, improving memory deficits of transgenic mouse models of AD [61]. Even though there is a lack of prospective and controlled studies, the overall available evidence favors the good cognitive tolerability of VPA.

The precise mechanism through which **GBP** exerts its therapeutic effects is not well understood. GBP binds the α2δ-1 subunit of voltage-gated calcium channels, which promotes the transfer of pore-forming α1 subunits of calcium channels from the endoplasmic reticulum to the cell membrane of pre-synaptic neurons. These subunits play a pivotal role in the pathophysiology of hyperalgesia. Chronic pain increases the expression of α2δ subunits and leads to the development of hyperalgesia [62]. Most studies stress the safety of GBP on cognitive functions, showing only minor or no adverse effects [63,64,65]. In contrast, just a few underpowered studies reported a possible worsening in attention, verbal memory, and general executive functioning [66,67] after GBP chronic treatment. In addition, GBP showed neuroprotective properties, especially in preclinical models of cerebral ischemia, through activation of the PI3K/Akt/mTOR pathway. The resulting antioxidant and anti-autophagic effects prevent neuronal loss after ischemic injury [68]. 

Further ASMs, namely pregabalin (PGB), lamotrigine (LMT), levetiracetam (LEV), and ZNS, have been occasionally described as therapeutic options for migraine prophylaxis. **PGB** is a pre-synaptic voltage-gated calcium channels inhibitor that modulates the release of several neurotransmitters involved in pain signaling, including glutamate, substance-P, norepinephrine, and calcitonin-gene-related peptide [69]. In addition, PGB increases the threshold for CSD, showing a specific mechanism of action against migraine [70]. PGB negatively impacts cognition, especially visuospatial memory, processing speed, and attention [71], although the effect is generally mild-to-moderate and lower than other ASMs. On the other hand, PGB has shown neuroprotective effects in experimental models of diabetes mellitus by reducing oxidative damage and showing anti-apoptotic properties [72]. However, testing PGB in an animal model of AD has provided poor results, questioning the use of the compound as a possible therapy in neurodegenerative disease [73].

**LMT** has positive effects on cognition and exhibits neuroprotective properties [74]. LMT, a selective inhibitor of voltage-gated sodium channels, also inhibits presynaptic glutamate and aspartate release [75]. LMT treatment reduced the number and size of amyloid plaques in the brain and increased the concentration of BDNF and nerve growth factor (NGF) in a preclinical model of AD [76]. Similar positive effects have been described for **LEV**, which also improves a range of cognitive domains, including attention and short-term and working memory [77,78]. AMPA receptor blockade explains the neuroprotective properties of the compound, especially in AD patients [79]. On the contrary, **ZNS** has been associated with cognitive profile worsening and the onset of attention and memory deficits [80]. ZNS is a sulfamate compound that blocks sodium and T-type calcium channels, which share common CAEs with topiramate [55]. ZNS reduced amyloid deposition and tau phosphorylation in a mouse model of type-2 diabetes [81].

**Conclusions:** ASMs may be very effective for migraine prevention, but they carry more side effects than other drug classes. Topiramate has the heaviest impact on cognition. Of note, other ASMs proposed for migraine prophylaxis, such as LMT and LEV, show better tolerability profiles and might be considered for migraineurs at risk for cognitive impairment (e.g., positive family history).

### 3.2. Antidepressants

Antidepressants are a broad category of drugs primarily used to treat mood disturbances. Despite poorly understood mechanisms of action, their effectiveness for migraine treatment is also acknowledged. The impact of long-term therapies with serotonergic drugs on the brain’s amyloid load has been investigated with positron emission tomography (PET) brain scans. Protective effects on amyloid deposition and a correlation with delayed conversion time from mild cognitive impairment (MCI) to AD (72) have been reported. Specific antidepressants are detailed below.

#### 3.2.1. Tricyclic Antidepressants 

**Amitriptyline** is a tricyclic antidepressant (TCA) that acts as a mixed serotonin–norepinephrine reuptake pump inhibitor (SNRI), sodium-channels blocker, and has antimuscarinic and antihistaminic effects [82]. It is effective for migraine prevention at a lower dose than those used in depression, even in the absence of mood disorders [82]. The mechanism by which amitriptyline prevents migraine attacks relies on its activity as an endogenous pain modulator and suppressor of CSD [83]. Chronic treatment with amitriptyline inhibits CSD by reducing sodium channel synthesis, desensitizing presynaptic receptors, and producing long-lasting monoaminergic neurotransmission changes [84]. Amitriptyline also modulates the noradrenergic descending inhibitory systems, including α2-adrenoceptor in the spinal dorsal horn, by activating noradrenergic neurons in the locus coeruleus and enhancing noradrenergic signaling [85]. Overall, clinical studies revealed detrimental effects on cognition, which are most prominent in elderly subjects and MCI patients [82,86,87,88]. Cognitive side effects of amitriptyline are unspecific, but a correlation with non-amnestic MCI (naMCI) has been posited since the anticholinergic and antihistaminic effects primarily affect attention, decision-making, and psychomotor speed [86,87]. Memory deficits are less frequent and appear at higher doses [86].

Due to its anticholinergic properties, a substantial side effect is the worsening of cognition, as confirmed by a German retrospective study on amitriptyline-treated subjects with pre-existent cognitive impairment [87]. Amitriptyline is classified as a third-class drug (the highest) on the Anticholinergic Cognitive Burden scale [87]. Anticholinergic agents trigger cognitive decline by reducing neuronal connectivity from the basal forebrain to the hippocampus and cortex [88]. The antihistaminic properties also promote cognitive impairment by inducing sedation (7) through H1 receptors [89]. 

On the other hand, neuroprotective properties of amitriptyline have been observed. Amitriptyline modulates neurotrophin levels [90] and acts as a TrkA and TrkB receptor agonist, thereby showing beneficial effects by blocking neuronal death and hippocampal apoptosis [90]. Similarly, in a rotenone rat model of PD, amitriptyline administration increased BDNF levels and promoted dopaminergic neuron survival [91]. 

#### 3.2.2. Serotonin and Norepinephrine Reuptake Inhibitors and Selective Serotonin Reuptake Inhibitors

Serotonin and norepinephrine reuptake inhibitors (SNRIs) are antidepressant drugs frequently employed in clinical practice [92]. These medications are also used for migraine prevention and are particularly useful in comorbid depressed patients [83,93]. The mechanism of action is related to modulation of descending pain pathways, mainly due to the potentiation of norepinephrine transmission [92,93]. SNRIs—particularly duloxetine—are highly effective as anti-migraine drugs in subjects showing “descending pronociceptive” features [92]. Pronociceptive migraineurs are less responsive to NSAIDs than centrally acting pain modulators [92]. 

**Venlafaxine** is an SNRI with a high safety profile, even in the elderly, due to no affinity for adrenergic, muscarinic, or histaminergic receptors [94,95]. Venlafaxine was found as effective as amitriptyline for migraine prevention [96] by modulating descending pain pathways [92]. Little is known about the effects of venlafaxine on cognition. However, results from preclinical studies indicate neuroprotective activity [92,97,98] in terms of myelin integrity [97] associated with improved working memory performances. This finding was not correlated with mood symptom improvement [97].

**Duloxetine** is an SNRI also effective for migraine prevention, especially in depressed and fibromyalgia patients [83,93]. Preclinical models showed that duloxetine exhibits antioxidant properties that can counteract free radical generation and apoptosis [92], as well as anti-glutamatergic and pro-cholinergic actions [99]. In chronic immobilization stress-induced mice, duloxetine inhibited hippocampal degeneration by up-regulating BDNF-driven neurogenesis, synaptic plasticity [99], and neurite outgrowth [100]. In depressed patients, the clinical effects of duloxetine on cognition are positive. Duloxetine improved attentional deficits in PD [92], as well as behavioral symptoms and memory deficits [92,101]. The compound also improves psychomotor processing, set-shifting, and working memory [101]. Of note, the positive effects were also found in non-depressed subjects [101]. In conclusion, duloxetine seems effective in promoting neuroprotective and precognitive effects.

Although selective serotonin reuptake inhibitors (SSRI) are a second-line migraine treatment, they effectively reduce the frequency and pain intensity of monthly episodes [83,93]. **Fluoxetine** blocks serotonin reuptake in the synaptic cleft and has an inhibitory effect on 5-HT_2C_ receptors [83,93]. Higher doses also increase norepinephrine levels and modulate central opioid receptors [83,93]. The compound promotes hippocampal neurogenesis [102,103] and has neuroprotective effects by increasing BDNF levels [104,105] and counteracting amyloid-driven neuronal toxicity [106,107]. Fluoxetine supplementation is protective in neuronal cultures challenged with synthetic human amyloid oligomers. A small randomized placebo-controlled trial in non-depressed MCI patients showed that 8 weeks of fluoxetine treatment improves performances on the MMSE test and immediate and delayed logical memory tests [108].

**Escitalopram’s** effect on amyloid load has been tested on APP/presenilin 1 mice. The compound significantly reduced (about 30%) the amyloid plaque load in a dose-dependent fashion by increasing the expression of α-secretases and thus enhancing the non-amyloidogenic pathway [109]. In humans, escitalopram administration was associated with mild, non-significant, short-term verbal memory deficits that worsened and became significant after pindolol (5-HT_1A_ partial agonist) add-on administration. Furthermore, spatial working memory deficits were observed upon intake of escitalopram + ketanserin (a 5-HT_2A_ antagonist) [110], thus highlighting that serotonin pharmacological modulation could affect performances at memory tests. According to the authors, high CNS levels of serotonin could induce cognitive impairment as well as low levels in a bell-shaped curve [110]. These findings align with the notion that, in PD patients, administration of pro-serotoninergic medications leads to better cognitive trajectories [111], possibly due to restoring normal serotonin levels after the neurodegenerative depletion of serotonergic transmission. The impact of SSRI and SNRI medications may differ according to subjects’ previous serotonin levels. 

**Conclusions**: All the anti-depressants seem to have a favorable biological impact in terms of neuroprotection. Nonetheless, amitriptyline—deemed the most effective compound for migraine prevention—carries significant CAEs due to its anti-cholinergic and anti-histaminergic properties. The serotonergic asset must be considered before prescribing anti-depressants for migraine prevention. 

### 3.3. Beta-Blockers

Several antihypertensive drugs are effective in slowing cognitive decline [112], although it remains unclear if the protective effects observed are linked to cardiovascular protection or modulation of neurodegenerative processes. Beta-blockers are the first-line drugs for hypertension [113]. Propranolol, metoprolol, atenolol, and bisoprolol are also first-line preventive therapies for migraine [20]. Although the use of beta-blockers is common in elderly individuals, it is still unclear whether this class of drugs impacts the development of cognitive conditions. Studies on beta-blockers as a group mostly found, in elderly subjects, an increased risk of vascular dementia (after correction for confounders, such as atrial fibrillation, incident coronary event, stroke, and heart failure) but not for all-cause, AD, or mixed dementia [114]. However, these results primarily focus on older adults taking beta-blockers to manage hypertension. Thus, it is likely that many other confounders could interfere. Of note, only long-term use of beta-blockers affects cognition [115]. Nonetheless, each compound has a different capacity for crossing the blood–brain barrier (BBB) and thus may differently impact the CNS. On one hand, cognitively impaired subjects taking CNS-active beta-blockers exhibited a consistent trend to worsen memory retrieval and MMSE scores when compared to untreated patients [116]. On the other hand, highly BBB-permeable compounds (e.g., propranolol or carvedilol) decreased the risk of developing AD compared to low permeable compounds (e.g., atenolol, sotalol, or bisoprolol) according to a large nation-wide retrospective cohort from Denmark [117]. The effect was significant after a minimum of 1.5 years of treatment and was attributed to improved clearance of AD-related neurotoxic molecules [117]. Since each compound has unique biochemical properties, a separate evaluation of each anti-migraine beta-blocker is still warranted. 

**Propranolol** is a lipophilic non-cardioselective beta-blocker with membrane-stabilizing properties [118]. Besides managing hypertension, angina pectoris, myocardial infarction, and cardiac arrhythmias [118], propranolol is also used as migraine prophylaxis [20]. The noradrenergic system is essential in modulating memory processes and inhibiting beta-noradrenergic receptors interfering with emotional memory reconsolidation [119]. Accordingly, propranolol has been used to treat post-traumatic stress disorder and is most effective when administered before extinction of the stimulus. This evidence has shifted the attention to the existence of a propranolol-driven cognitive bias that affects decision-making [120]. Furthermore, compared to atenolol (a hydrophilic compound), a single dose of propranolol increased the manipulation costs of a working memory task in young subjects [121]. However, no differences in terms of memory maintenance were observed. More comprehensive clinical investigations revealed no significant cognitive and psychological alterations in propranolol-treated patients with hypertension [122,123,124], except for one study showing mild long-term impairment in sustained attention [124]. 

Propranolol has shown neuroprotective effects in a preclinical AD model by reducing Aβ1-42 hippocampal accumulation and decreasing tau hyperphosphorylation through modulation of the GSK3β/JNK1 pathway [125]. Propranolol also inhibits Aβ1-42-driven increase in cAMP levels and decreases ApoE expression [125]. This evidence suggests the potential association between propanol and ApoE homeostasis in astrocytes, which could counter Aβ neurotoxicity.

**Metoprolol** is a selective beta1-adrenergic antagonist with moderate BBB permeability, recommended for episodic migraine prevention [126]. Metoprolol reduces cardiovascular events and mortality in patients with hypertension and coronary heart disease [127]. From a cognitive viewpoint, metoprolol improves proofreading, visual–motor performance, and several measures of complex managerial competence [128]. However, metoprolol has also exhibited immunomodulatory effects, increases pro-inflammatory conditions, and attenuates anti-inflammatory signaling [129]. In addition, chronic use of metoprolol, while impairing synaptic phagocytosis, potentiated synaptic degeneration and acceleration of neurodegeneration in CNS disorders [129]. Whereas the pro-inflammatory effects of acute beta-blocker administration have been observed only in the CNS in the amyloidosis model, chronic effects of metoprolol warrant additional research with potential relevance to human health.

**Atenolol** is a hydrophilic, generally well-tolerated selective adrenoceptor antagonist [130]. Unlike propranolol, atenolol treatment does not significantly affect cognitive performance [131,132]. However, two case reports demonstrated cognitive improvement after atenolol withdrawal [133]. Finally, atenolol treatment may be associated with a higher number of errors in a visual–motor task and with impairment in one measure of complex management [134].

**Bisoprolol** is a long-acting selective blocker of beta1-adrenergic receptors used for management of congestive heart failure and prevention of episodic migraine attacks [135]. Despite its widespread use in clinical practice, there is no evidence linking the drug to cognitive impairment or neurodegeneration.

**Conclusions**: The effect of beta-blocker administration on cognition and neurodegenerative processes seems more controversial than other drug classes, and further studies are needed.

### 3.4. Calcium Channel Blockers 

According to the Italian guidelines for primary headaches, the calcium channel blockers cinnarizine and flunarizine are recommended as preventive treatments for disabling migraine [21]. However, the use of these drugs, especially in elderly patients, often results in common side effects such as drowsiness, sedation, weakness, and depression [136]. In addition, they may induce extrapyramidal symptoms and drug-induced Parkinsonism (DIP) [137] due to their pre-synaptic and post-synaptic effects. In particular, the D2 receptor blockade and the loss of tyrosine hydroxylase in the monoaminergic presynaptic can lead to movement disorders [138,139]. Less well-known are the neurocognitive effects of these drugs, particularly their long-term tolerability. 

Calcium acts as a second messenger and regulates numerous biological processes, such as muscle contraction, neuronal plasticity, cell migration, cell growth, and excitability [140]. Calcium is also implicated in releasing neurotransmitters, including glutamate, via voltage-gated channels (N-type and P/Q-type) located at the presynaptic terminals. Once released, glutamate promotes calcium entry through NMDA receptors and, indirectly, through L-type calcium channels. Excessive glutamatergic stimulation can lead to excessive ion flux into the cell (particularly sodium and calcium), resulting in oxidative stress, neuronal damage, and subsequent cell death [141,142,143,144]. This process, termed excitotoxicity, participates in many neurodegenerative disorders [145], including headache, epilepsy, AD, and vascular disorders [144,146].

**Cinnarizine** is a calcium channel blocker (CCB) that inhibits vascular smooth muscle cell contractions by blocking voltage-gated L- and T-type calcium channels. Cinnarizine antagonizes dopamine D2 receptors, histamine H1 receptors, and muscarinic acetylcholine receptors (weak anticholinergic activity) [147]. Antihistaminic and antimuscarinic drugs are known to impair cognitive processes [148], but D2 antagonism also plays an important role in impairing working memory [149]. The dopaminergic system is involved in memory processes and the proper functioning not only of the hippocampus but also the striatal (caudate) dopaminergic system, intermingled in numerous cognitive networks [150,151]. 

Cases of cinnarizine overdose have been reported in pediatric and adult children [152], with effects including a range of symptoms such as drowsiness, coma, vomiting, hypotonia, stupor, and seizures. In addition, a study reported that, when compared to the pre-intake status, cinnarizine administration significantly impaired psychomotor performance, information processing, and vigilance [153]. The maximum effect occurred 5–6 h after drug administration and lasted for 10 h, an effect linked to drug-mediated blockade of CNS H1 receptors. However, a recent study showed that cinnarizine does not affect psychomotor speed, attention, vigilance, and working memory [154].

Other studies reported no adverse effects or even a protective role of cinnarizine. A placebo-controlled trial on pediatric PwM [155] showed no serious adverse effects. No cognitive adverse effects were reported—but neither specifically assessed. Finally, in a preclinical model of haloperidol-induced cognitive impairment, cinnarizine reduced brain oxidative stress and prevented memory deficits [156]. 

**Flunarizine** is a selective CCB with calmodulin-binding properties also exhibiting histamine H1 blocking activity. Flunarizine has structural and pharmacological commonalities with cinnarizine [157]. However, it has a longer plasmatic half-life and can be used in a single daily administration. Flunarizine reduces excessive calcium entry into the cell, thereby preventing cation overload without affecting physiological calcium homeostasis [158,159]. Flunarizine reduces systemic oxidative stress in patients with migraine. It also regulates changes in cerebral blood flow, thus protecting the cerebral endothelium from oxidative insults [158]. Through its antioxidant potential, it plays a role in blocking membrane lipid bilayer peroxidation [160]. Finally, flunarizine also modulates cholinergic transmission by promoting acetylcholine (Ach) release and by altering the expression/affinity of muscarinic receptors [161,162]. 

Flunarizine also relieved cerebral ischemia-reperfusion-related cognitive and motor deficits through increased tissue calcium and AchE level [163]. The effect was due not only by the calcium channel blockade, but also antioxidative, lipid anti-peroxidation, and anticholinesterase activities. 

Flunarizine is generally well tolerated and rarely causes major side effects. The main adverse effect experienced by patients is drowsiness, which can be avoided by slow titration of the drug and taking it in the evening [164]. Few clinical studies are available on the specific effect of flunarizine on cognitive function. A randomized controlled trial of flunarizine as adjunctive ASM in children with infantile spasms [165] did not reveal a protective effect of flunarizine on cognitive outcomes. However, subgroup analysis indicated that it might further improve cognitive outcomes in children with no clear disease etiology. 

**Conclusions**: Calcium channels blockers carry important side effects that can, indirectly, impair cognition (e.g., sedation and depression) and motor abilities (DIP). Despite limited evidence suggesting potential neuroprotective effects, the anti-histaminergic and anti-dopaminergic CAEs seem to prevail.

### 3.5. Inhibitors of the Renin–Angiotensin System (RAS)

**Candesartan** is an anti-hypertensive medication acting as an angiotensin (AT) II receptor blocker (ARB). The primary anti-migraine mechanism of action is related to modulation of the intrinsic RAS of the CNS, which is largely independent of the systemic one [166], and to the inhibition of nitric oxide release by microglia [167]. Activation of the AT receptors is a risk factor for AD; therefore, central-acting RAS inhibitors might represent promising pharmacological aids [168]. The hypothesis is supported by the observation that MCI individuals receiving RAS inhibitors show slower cognitive decline [169] and a reduced risk of developing AD [170]. The protective effect is mediated by the anti-inflammatory activity of the compound, exerted through AT receptor blockade. Mechanistically, candesartan inhibits the release of inflammatory mediators, such as TNF-α and TGF-β1, and counteracts the expression of COX-2 and iNOS [167]. In a preclinical AD model, intranasal administration of candesartan reduces Aβ deposition [167]. Interestingly, the effect is unrelated to changes in Aβ metabolism but occurs through enhanced microglial phagocytosis of Aβ deposits. Other ARBs (irbesartan and telmisartan) also display promising anti-inflammatory features, primarily driven by agonism of the transcription factor PPARγ, and could also be associated with reduced risk of diabetes [171,172], a known predisposing factor for cognitive impairment. Candesartan exhibited anti-inflammatory and anti-neurodegenerative properties also in a post-stroke experimental model [173], possibly preventing mixed forms of dementia. Administration of candesartan was not only associated with a reduced degree of reactive post-ischemic gliosis but also with reduced deposition of β-amyloid in dense hippocampal plaques and reduced Aβ_1-42_-related toxicity [173]. A modest anti-amyloid effect was also documented, after candesartan treatment, in mice carrying the Swedish or Indiana APP mutation compared to untreated animals [174]. In the same experiment, candesartan significantly decreased APP-related neuroinflammation, as expressed by GFAP-positive astrocytes and Iba-1-activated microglial cells, and promoted hippocampal neurogenesis by increasing BDNF levels [174]. 

From a clinical viewpoint, candesartan administration did not show a major effect on global cognition, assessed by MMSE, in the SCOPE (Study on Cognition and Prognosis in the Elderly) trial, conducted on elderly subjects with hypertension [175]. However, a sub-study showed that treated patients had better performances in terms of attention and episodic memory, whereas no longitudinal differences were observed in executive functions, working memory, and cognitive speed [176]. The HOPE3 clinical trial, however, where candesartan was administered with hydrochlorothiazide (HCT), showed that scores on the Montreal Cognitive Assessment (MoCA), Digit Symbol Substitution Test (DSST), or Trail Making Test part B (TMT-B) did not differ between the candesartan + HCT group and the placebo group [177]. However, when administered alone, candesartan improved executive functions in MCI subjects [178,179]. Candesartan has recently landed in one phase-two double-blind placebo-controlled randomized clinical trial (CEDAR—Candesartan’s Effects on Alzheimer’s Disease and Related Biomarker), in which MCI subjects were administered the maximum tolerated dose of the drug [180]. Although the enrollment phase was concluded, the results are still not available.

**Conclusions**: Compared to other migraine preventive treatments, including beta-blockers, candesartan seems to show the most promising profile to counteract neurodegeneration and neuroinflammation-driven cognitive impairment.

### 3.6. Anti-CGRP and Gepants

The calcitonin-gene-related peptide (CGRP) is a 37-aa neuropeptide ubiquitously expressed from alternative splicing of the *CALCA* gene, mostly in the peripheral nervous system and CNS [181]. CGRP regulates smooth muscle cells of the peripheral vasculature and also modulates vasodilation through non-endothelial mechanisms by activation of adenylate cyclase and generation of intracellular cyclic adenosine monophosphate [182]. CGRP modulates the neuronal activity in the trigeminocervical complex [183] and the transmission of pain signals to the thalamus and cortical brain regions [184]. The role of the trigeminal system and CGRP is profoundly intertwined in the pathophysiology of migraine, as confirmed by the expression of CGRP receptors on trigeminal C- and A- nociceptive fibers. Moreover, CGRP is known to be released into the cranial venous system during migraine attacks. 

These findings led to the development of anti-migraine therapies that inhibit CGRP action. Small-molecule α-CGRP receptor antagonists, gepants, and fully humanized monoclonal antibodies towards CGRP and the α-CGRP receptor showed beneficial effects in reducing migraine symptoms in clinical trials [19,185]. The **Gepant** class now includes ubrogepant and rimegepant [19], whereas **monoclonal antibodies** against CGRP include fremanezumab, galcanezumab, and eptinezumab [185]. Only one monoclonal antibody directed against the receptor complex (erenumab) is commercially available [185]. While gepants are approved as abortive drugs that reduce attack frequency [19], monoclonal antibodies are considered third-line prophylactic therapies [185]. Monoclonal antibodies and gepants have only recently been made commercially available, and there is now limited evidence on the long-term effects of the drugs. 

CGRP is physiologically expressed in brain regions connected to memory, attention, autonomic, and behavioral functions [186]. Many preclinical studies indicate that the peptide profoundly affects behavior [187,188] and biological processes related to mood and cognition. CGRP, by enhancing IGF-1 production, promotes hippocampal neurogenesis and synaptic plasticity, resulting in improved spatial learning performances in mice [189,190,191]. An analog effect has been reported after donepezil administration [192]. Overall, several observations point towards CGRP involvement in neuroprotection in different conditions, including ischemia-reperfusion injury and experimental allergic encephalomyelitis [193,194]. CGRP also activates amylin type 1α receptors, and amylin is thought to play a protective role in AD [195,196]. Accordingly, lower CGRP concentrations in the cerebrospinal fluid (CSF) and worse neuropsychological profiles, albeit in specific domains, such as selective attention and visuoperceptual functions, have been reported [186].

CGRP has also been suggested to participate in AD pathogenesis. In a preclinical AD model, the 5XFAD mouse, treatment with an α-CGRP receptor blocker (BIBN) reduced the pathology-related phenotype [197] by enhancing the expression of PSD95, a synaptic protein involved in synaptic plasticity [198]. BIBN administration also decreased α-synuclein expression and aggregation in the AD mouse model. Finally, BIBN reduced the cortical and hippocampal Aβ burden and tau phosphorylation [197]. Inhibition of the α-CGRP receptor counteracted the pro-inflammatory p38-MAPK-NFκB pathway, supporting the CGRP role in inflammation. The peptide exerts a bimodal effect on the immune system by exhibiting anti- and pro-inflammatory properties [199,200]. However, the pro-inflammatory effects in neurological disorders such as migraine are more prominent [201,202].

No cognitive side effects have been reported with antagonists of the CGRP system. Of note, monoclonal antibodies do not cross the BBB [203], whereas gepants have limited access [204] and could exert some activity on the CNS. 

**Conclusions:** Most studies suggest a neuroprotective role for the peptide. However, some conflicting evidence from a preclinical model prompts further research to confirm the hypothesis.

### 3.7. Triptans and Ditans

**Triptans** are selective 5-hydroxytryptamine_1B/1D_ (5-HT_1B/1D_) receptor agonists used as abortive drugs for migraine attacks, usually when FANS are ineffective [20]. Triptans (sumatriptan, zolmitriptan, almotriptan, eletriptan, frovatriptan, naratriptan, and rizatriptan) have replaced ergot-derived compounds [205]. Both 5-HT_1B_ and 5-HT_1D_ receptors are expressed within the trigeminal nerve and ganglion. They counteract migraine-related vasodilation through different mechanisms [205]. Further, 5-HT_1B_ receptors are localized within smooth muscle cells of blood vessels and inhibit vascular contraction [205]. The process is also facilitated by pharmacological agonism on 5-HT_1D_ receptors. These are located in the efferent trigeminal fibers that innervate the dural vessels and inhibit release of vasoactive peptides [205]. Despite some initial controversies [206], triptans can effectively cross the BBB and influence the expression of serotonergic receptors [206,207]. 

In physiological conditions, 5-HT_1B_ receptors are most densely expressed within the thalamus (especially in the ventromedial nucleus, VMN) and the basal ganglia, followed by the cerebral cortex and, in particular, within the precentral and postcentral gyri [208]. Further, 5-HT_1D_ receptor expression shows several topographical overlaps (thalamus and basal ganglia), but it is also expressed within the midbrain (dorsal and medial raphe nuclei) [208]. At the cortical level, 5-HT_1D_ receptors are mainly located within the piriform cortex [208]. The VMN is a primary target for nociception control and acts as a gate for the transmission of noxious stimuli to the cortex [209]. Furthermore, modulation of VMN neurons can have a significant and widespread impact on cortical activity [210], particularly the frontal cortex [211], thanks to their extensive connections.

Alterations in the serotonergic system, including a significant reduction in 5-HT_1B/1D_ receptors, were found post-mortem in AD brains compared to controls [212]. The reduction was observed in the prefrontal (BA10, 25%) and temporal (BA20, 37%) cortices [212]. Expression levels of 5-HT_1B/1D_ receptors in the frontal lobe positively correlate with cognitive decline in the patient’s lifetime. In contrast, no differences in receptor affinity for the ligand were observed [212]. This is not surprising as BA10 has a key role in memory retrieval [213]. As 5-HT_1B/1D_ receptors at pre-synaptic terminals inhibit Ach release, their downregulation could represent a compensatory mechanism [212,214,215,216]. Accordingly, the agonism on 5-HT_1B/1D_ receptors—such as the one exerted by triptans—could impair memory consolidation processes [217,218,219,220,221]. While in vivo studies documented a down-regulation of 5-HT_1B_ receptors after administration of sumatriptan in migraine, effects on cognition have not been thoroughly investigated [207]. 

From a clinical viewpoint, memory impairment is listed among the potential (infrequent) neurological side effects of rizatriptan [222]. Anecdotic reports suggest that acute or chronic exposure to triptan-driven vasoconstriction induces transient global amnesia-like symptoms, possibly due to reversible ischemia [223,224]. Preliminary results also suggest reversibility of 5-HT_1B/1D_-driven memory impairment by administering 5-HT_1B/1D_ antagonists and ultimately restoring cholinergic-driven learning consolidation [221,225,226]. Further studies are needed to assess the actual effectiveness and safety of these compounds. However, it is important to remember that sumatriptan administration restores cognitive functioning when disturbed by migraine-related pain, providing short-term benefits [227].

A new class of drugs has been recently approved, **ditans**, with prototypal drug lasmiditan [19]. Ditans activate trigeminal 5-HT_1F_ receptors and block the release of CGRP and other vasoactive peptides. Ditans do not affect 5-HT_1B_ receptors and, therefore, do not directly promote vasoconstriction, thereby limiting the cardiovascular- and cerebrovascular-related side effects [228]. Interestingly, 5-HT_1F_ agonists such as lasmiditan block the release of glutamate and neuronal hyperexcitability, a process related to pain sensitization and AD neurodegeneration [228]. Lasmiditan has easy access to the CNS due to the high lipophilicity of the molecule and, although it may provoke somnolence or fatigue, no clear-cut adverse cognitive effect has been reported [229], possibly due to a relatively low expression of 5-HT_1F_ receptors in the brain tissue [208]. Interestingly, the hippocampus is one of the regions more enriched in 5-HT_1F_ receptors [230]. Thus, any potential drug effect in terms of neuroprotection would be exerted in a key structure for memory processes.

**Conclusions**: The current evidence suggests a potential null-to-positive effect for lasmiditan on neurodegenerative pathways and cognition, whereas triptans show potential interference with memory processes due to presynaptic inhibition of Ach release. 

### 3.8. Ergots

**Ergotamine** and **dihydroergotamine** (DHE) efficacy profiles are similar to triptans. However, these compounds are progressively out-phased due to the more frequent adverse events and cases of abuse [20,21]. To date, DHE is rarely employed for severe refractory attacks that are unresponsive to other abortive medications, whereas ergotamine or other ergots carry lower levels of indication [19,21]. Ergot alkaloids bind—as agonists—5-HT_1B/1D_ receptors, such as triptans, 5-HT_1F_-like ditans with lower affinity, D_2_ dopaminergic, and α-adrenergic receptors [231]. Ergots exert anti-migraine activity by promoting intense vasoconstriction and interfering with the trigeminovascular system [231]. 

Increased latency of the N2 component in an event-related potentials (ERP) study indicated potential impairment in cognitive processes and pre-attentive stimulus evaluation about two hours after ergotamine intake [232]. The finding was attributed to changes in the “automatic memory updating” system that participates in the earliest stages of cognitive processing for unpredictable stimuli, possibly relying on abnormal hippocampal functioning [232]. Furthermore, in ergotamine abusers, a significant cognitive impairment was documented for complex reaction time tasks, cognitive flexibility tests, and verbal memory [233]. However, the dose-dependency of the effect still needs clarification.

Overall, the clinical evidence indicates a detrimental effect of ergotamine on cognition, but the actual impacts of ergots on neurodegenerative or neuroprotective pathways are still inconclusive. For instance, a previous study evaluating the potential neuroprotective effect of alkaloid derivates in AD [234] found that dihydroergocristine, but not ergotamine or dihydroergotamine, inhibits γ-secretase and interferes with β-amyloid deposition [234]. Further evidence indicates that plasma levels of Aβ_1-42_ do not differ in ergot-users [235], but it should be stressed that this measure is not an established biomarker for AD and is only used for research purposes [16]. Furthermore, drug-driven cognitive impairment may depend upon different neurodegenerative pathways that have not been explored yet.

**Conclusions**: The use of ergots should be limited to selected cases due to several adverse effects, including potential CAEs.

### 3.9. Non-Steroidal Anti-Inflammatory Drugs (NSAIDs)

Several studies have investigated the potential effects of NSAIDs on neurodegeneration. NSAIDs’ mechanism of action depends primarily on the inhibition of cyclooxygenases that convert arachidonic acid into pro-inflammatory mediators, such as prostaglandins, prostacyclins, and thromboxanes. The application of COX-inhibitors to migraine treatment is mainly due to the inhibition of neurogenic inflammation and trigeminal nociception, as well as modulation of the brainstem and thalamic antinociceptive systems [236]. Growing evidence indicates that neuroinflammatory processes contribute to AD pathophysiology [237,238], suggesting that NSAID-based anti-inflammatory interventions are neuroprotective [239,240].

Several NSAIDs (e.g., nimesulide, indomethacin, and ibuprofen) up-regulate the expression of α-secretase, promoting the non-amyloidogenic processing of APP [241]. Similarly, compelling evidence indicates that NSAIDs can lower Aβ_1-42_ levels in vitro and in vivo by modulating γ-secretase cleavage activity [242,243]. NSAIDs also act downstream from Aβ_1-42_ metabolism by inhibiting the formation of amyloid fibrils and destabilizing those already aggregated [244]. 

Conflicting evidence has been found while evaluating NSAIDs in clinical settings. While many observational studies supported the protective role of NSAIDs in AD [240,241], other studies showed opposite results [245,246,247].

On a final note, the Food and Drug Administration has recently allowed one phase-three study on rofecoxib (COX-2 inhibitor) as an acute treatment for migraine attacks [248]. Close monitoring of cerebrovascular adverse events is warranted due to a previous report about an increased risk of ischemic stroke upon taking this medication [249] and the higher stroke risk in migraineurs (see Section 2.1). 

**Conclusions**: The biological activity of NSAIDs suggests an overall neuroprotective role, confirmed by some clinical observations. The conflicting results may depend on the intra-class variability among the compounds’ features or the clinical trial designs.

## 4. Discussion

### 4.1. Migraine and Dementia—The Missing Link

Observational studies and meta-analyses support the idea that migraine attacks and their frequency are associated with risk of dementia. However, the combined effects exerted by different classes of anti-migraine compounds on cognition and neurodegeneration are still largely unexplored. As summarized in Table 1, some anti-migraine drugs display an overall positive effect, others negatively impact dementia-related pathologies, and a few exhibit conflicting properties (e.g., they are neuroprotective but also impair cognitive processes). 

**Figure 1:**
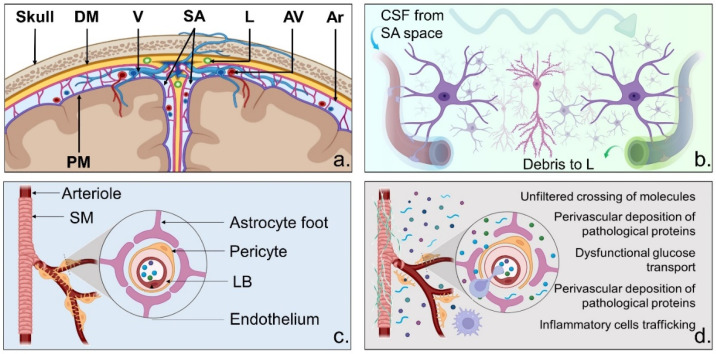
The missing link. The figure depicts the anatomy and physiology of the neurovascular and glymphatic systems. (**a**) Neuroanatomy of the meninges. (**b**) Schematization of the glymphatic system, showing the entry of CSF within the paravascular space, permeating towards the veins, where it is collected along with neurotoxic molecules and drained into dural lymphatics. Engulfment of this system induces accumulation of debris, including neuroinflammatory and nociceptive mediators and misfolded proteins. (**c**) Physiological composition of the blood–brain barrier (BBB). The layers properly filter the arterial blood, and only selected molecules may cross. (**d**) Disruption of the BBB and related pathological changes. Abbreviations: Ar = arachnoid; AV = arterial vessel; CSF = cerebrospinal fluid; DM = dura mater; L = lymphatics; LB = lamina basalis; PM = pia mater; SA = subarachnoid space; SM = smooth muscle; V = vein.

Additional investigation is needed. Promising mechanisms to be explored concern the disruption of BBB integrity (see Figure 1). Migraine-related nociceptive molecules can enter the BBB [250]. BBB breakdown is also an early event that precedes brain atrophy in AD and other neurodegenerative conditions [251]. Thus, BBB is a potential trait d’union between migraine and dementia. However, recent imaging studies of BBB integrity in migraineurs or in vivo evaluation of migraine-inducing substances on BBB permeability have challenged this notion [250]. The poor sensitivity of the employed methods might be the root of the lack of positive correlations. Improved imaging techniques and/or focus on alternative biomarkers could, in future studies, resolve the issue [250].

The functioning of the glymphatic system offers another intriguing link. The system encompasses a network of perivascular channels that, during sleep, clean the interstitial brain space, removing neurotoxic molecules and other debris [252]. 

The system’s impairment is implicated in headache disorders [253,254]. In particular, CSD-driven migraine aura is accompanied by a temporary closure of paravascular spaces and the build-up of nociceptive molecules [255]. The sleep-driven activity of the glymphatic system in migraineurs provides the rationale for the empirical observation that sleep halts headache attacks while insomnia increases their frequency [254]. 

Sleep disturbances and glymphatic system dysfunction are common features in neurodegenerative settings, including AD, where system impairment contributes to enhancing Aβ and tau pathology [256,257]. However, whether headache-related glymphatic alterations participate in AD pathogenesis is still unknown.

However, the meningeal lymphatic system can participate in the onset of a migraine attack [258,259] by carrying nociceptive molecules [260]. Although poorly investigated, meningeal lymphatics ablation alters the balance of pro- and anti-inflammatory factors involved in migraine [260]. On the other hand, a growing body of evidence indicates that impairment in meningeal lymphatics and brain inflammation participate in AD pathogenesis [259,261].

### 4.2. Migraine Therapies, Cognitive Deficits, and Neurodegenerative Pathways—Final Remarks

**Figure 2:**
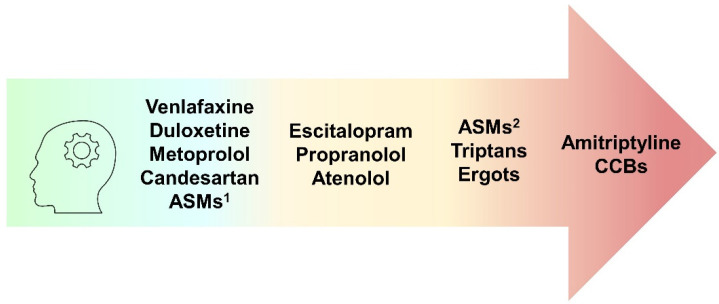
Cognitive effects of migraine treatments. In the green section: compounds with positive effects on cognition; in the yellow section: compounds with mild negative effects; in the orange–red section: compounds with the worst impact on cognitive performances. Abbreviations: ASMs = anti-seizure medications, type 1: lamotrigine, levetiracetam, type 2: valproic acid, topiramate, gabapentin, pregabalin, zonisamide, CCBs = calcium channel blockers.

Cognitive adverse effects may be triggered by migraine treatments with short latency through several mechanisms, including direct modulation of neurotransmitters’ systems. As illustrated in Figure 2, some ASM therapies, amitriptyline [86,87,88], and ergots [232,233] produce the heaviest burden on cognitive performances [45,46,47,48,49,50,57,58], along with calcium antagonists, which significantly increase sedation [137,165]. Escitalopram [110], propranolol [120,121], atenolol [134], or triptans [222] rarely generate mild symptoms. These iatrogenic symptoms may resemble naMCI features, whereas memory deficits are less common. Of note, positive effects are found after administration of SNRIs [92,97,101], metoprolol [129], or candesartan [176,178,179].

Long-term changes in cognition, however, may arise due to slower biological effects of the compounds on the CNS, which might play a role in the increased risk for late-onset dementia in some migraineurs. Metoprolol [129] was found to increase inflammation, whereas TPM [43,44], GBP [68], duloxetine [92], fluoxetine [107], cinnarizine [156], flunarizine, NSAIDs [236], and candesartan [167,173] either showed antioxidant capacities or favorably modulate the microglia–astrocyte axis. Neuroinflammation plays a pivotal role in AD and migraine pathogenesis [262] and represents a key therapeutical target. 

The “core” AD neurodegenerative processes (β-amyloid oligomers and fibrils in plaques, and accumulation of hyperphosphorylated tau in neurofibrillary tangles) [16] are directly modulated by VPA [61], fluoxetine [106,107], escitalopram [109], propranolol [125], candesartan [167,173,174], and NSAIDs [241,242,243,244]. 

Furthermore, active enhancement in the brain’s recovery from damage was found upon administration of TPM [44], amitriptyline [90,91], LMT [76], duloxetine [99,100], fluoxetine [104,105], or candesartan [174]. The drugs stimulate, through increased expression of BDNF and other neurotrophins, neurogenesis, which is increased in chronic degeneration to neutralize tissue damage [263]. Accordingly, long-term use of these compounds can offer substantial neuroprotective effects. 

**Figure 3:**
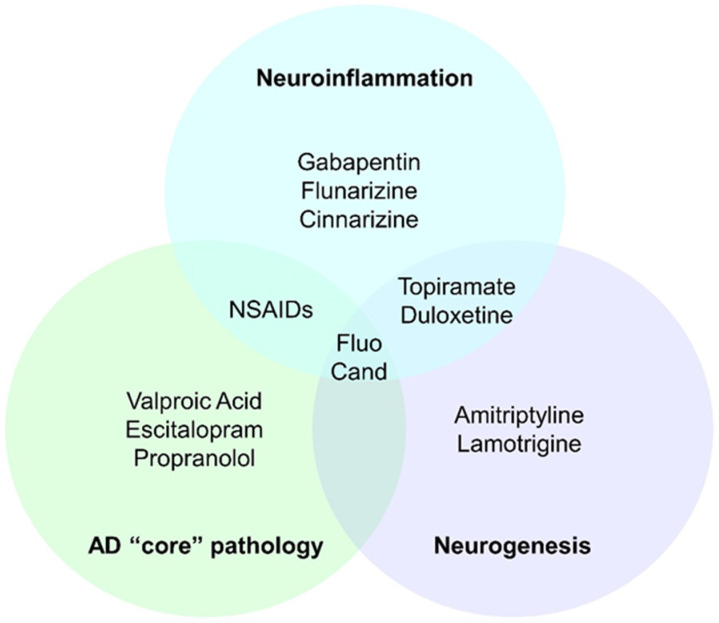
The diagram illustrates migraine therapies that positively impact CNS homeostasis by promoting neurogenesis or counteracting neuroinflammatory or neurodegenerative processes. Abbreviations: AD = Alzheimer’s disease; Cand = candesartan; Fluo = fluoxetine.

In conclusion, the anti-migraine efficacy of the compounds should be carefully balanced with the negative impact on the patients’ daily and work performances in the short-term and with potential neurodegenerative sequelae in the long term, especially given the availability of several therapeutical options that may counteract neurodegeneration and neuroinflammation (summarized in Figure 3). Future research should focus on the intersections shared by dementia and migraine to develop safe and possibly protective treatments.

**Supplementary Materials**: Features, study design, and main findings of the most relevant clinical trials are summarized in Supplementary Table S1. 

## Figures and Tables

**Table 1:** Summary of findings on the role of anti-migraine therapies on cognition and neurodegeneration.

Migraine Treatment	Indication	Biological Effect	Clinical Impact on Cognition	References
***ASMs***				
**TPM**	Prophylaxis	- ↑ Akt/GSK-3β/CREB pathway [§] - ↓ inflammation mediators (IL-1β, TNF-α) [§] - ↓ oxidative stress [§] - ↑ BDNF [§]	- mild-to-moderate impairment in attention, psychomotor abilities, language, and comprehension, including verbal fluency, short-term episodic and working memory, processing speed. Abnormal thinking. TPM is the ASM with the heaviest impact on cognition.	[41,42,43,44,45,46,47,48,49,50,51,52,53,54,55]
**VPA**	Prophylaxis	- ↑ taurine, glycine, serotonin, and dopamine in the hippocampus [§] - ↓ Aβ generation and tau hyperphosphorylation [#]	- mild attention, memory, and visuomotor deficits	[56,57,58,59,60,61]
**GBP**	Prophylaxis	- antioxidative and anti-autophagy properties by activating the PI3K/Akt/mTOR pathway [§]	- overall good tolerability. Few reports indicated deficits in attention, verbal memory, and executive functions after chronic use.	[62,63,64,65,66,67,68]
***Antidepressants***				
**Amitriptyline**	Prophylaxis	- antimuscarinic (↓ cholinergic pathways from the basal forebrain to the hippocampus and cortex) [◊] - antihistaminic [◊] - neuroprotection through TrkA and TrkB receptor agonism [#] and increase in BDNF levels [§]	- sedation - promotes cognitive impairment with a non-amnestic phenotype - impairs attention, decision-making, and psychomotor speed - memory deficits at higher doses	[82,83,84,85,86,87,88,89,90,91]
**Venlafaxine**	Prophylaxis	- enhances myelin integrity [#]	- improves working memory *	[92,94,95,96,97]
**Duloxetine**	Prophylaxis	- antioxidative effects [#] - anti-glutamatergic effects [#] - pro-cholinergic effects [#]	- improvement in neurodegeneration-driven attention, memory, and behavioral symptoms	[83,92,93,99,100,101]
**Fluoxetine**	Prophylaxis	- ↑BDNF levels and hippocampal neurogenesis [†] - ↓ amyloid neuronal toxicity through the expression of TGF-β1 and MMP-2 in the astroglia [§]	- improvement in global cognitive level (MMSE score), and immediate and delayed logical memory performances in MCI	[83,93,102,103,104,105,106,107,108]
**Escitalopram**	Prophylaxis	- ↑ α-secretase (non-amyloidogenic processing of APP) [#] - ↓ amyloid load [#]	- mild short-term verbal memory exacerbated by add-on of pindolol	[109,110,111]
***Beta-blockers***				
**Propranolol**	Prophylaxis	- ↓ of Aβ-driven cAMP levels and ApoE expression [#]	- cognitive bias in decision-making - ↑ working memory manipulation costs - no long-term evident changes in cognition or behavior	[118,119,120,121,122,123,124,125]
**Metoprolol**	Prophylaxis	- ↑ pro-inflammatory mediators [#] - ↓ anti-inflammatory mediators [#] - ↓ synaptic phagocytosis → synaptic degeneration [#]	- ↑ abilities in proofreading, visual–motor tasks, and complex management	[126,127,128,129]
**Atenolol**	Prophylaxis	- unknown	- does not significantly affect global cognition - impairs visual–motor performances and complex management components	[130,131,132,133,134]
**Bisoprolol**	Prophylaxis	- unknown	- Unknown	[135]
***Calcium-channel blockers***				
**Cinnarizine**	Prophylaxis	- anti-muscarinic, anti-histaminic, and anti-dopaminergic effects [◊] - antioxidant [#]	- sedation - conflicting results about the impact on psychomotor speed, attention, vigilance, and working memory	[147,148,149,150,151,152,153,154,155,156]
**Flunarizine**	Prophylaxis	- antioxidant [◊†] - ↓ lipid peroxidation [†] - modulates Ach transmission: ↑ Ach release [§] ↓ M receptor expression [§] ↑ M receptor affinity (Kd) [§]	- sedation, increased risk of Parkinsonism	[157,158,159,160,161,162,163,164,165]
***ARB***				
**Candesartan**	Prophylaxis	- ↓ release of neuroinflammatory mediators by microglial cells [§#†] - ↑ cell-mediated Aβ clearance [§#] - ↑ BDNF-driven hippocampal neurogenesis [#]	- positive effect on episodic memory and attention in cognitively normal elderly subjects - positive effect on executive functions in MCI subjects	[166,167,168,169,170,171,172,173,174,175,176,177,178,179,180]
***Monoclonal antibodies***				
**Erenumab, Fremanezumab, Galcanezumab**	Prophylaxis	- inhibition of CGRP-related effects, which seem mainly neuroprotective [◊]	- unknown (low CSF levels of CGRP have been linked to impaired selective attention and visuo-perceptual functions).	[19,181,182,183,184,185,186,187,188,189,190,191,192,193,194,195,196,197,198,199,200,201,202,203,204]
***Gepants***				
**Rimegepant,** **Ubrogepant**	Acute treatment	- inhibition of CGRP-related effects, which seem mainly neuroprotective [◊] - another receptor antagonist (BIBN) showed neuroprotective effects [#]	- unknown	[19,181,182,183,184,185,186,187,188,189,190,191,192,193,194,195,196,197,198,199,200,201,202,203,204]
***Triptans***				
**Almotriptan, Eletriptan, Frovatriptan, Naratriptan, Rizatriptan, Sumatriptan, Zolmitriptan**	Acute treatment	- agonism on 5-HT1B/D may impair cholinergic transmission [◊] - S administration → Down-regulation of 5-HT1B [◊]	- on rare occasions, memory deficits have been reported after R use.	[20,205,206,207,208,209,210,211,212,213,214,215,216,217,218,219,220,221,222,223,224,225,226,227]
***Ditans***				
**Lasmiditan**	Acute treatment	- via 5-HT1F: ↓Glu release [§]	- unknown	[19,228,229,230]
***NSAIDs***				
**Aspirin, ibuprofen, diclofenac, indomethacin, and others**	Acute treatment	- ↓ inflammation [◊] - ↑ α-secretase [†] - ↓ Aβ aggregation [†] - favorable modulation of γ-secretase [†]	- conflicting results in terms of protection/risk of AD.	[237,238,239,240,241,242,243,244,245,246,247]
***Ergots***				
***Ergotamine, DHE***	Acute treatment	- similar effects to triptans and ditans (modulation of 5-HT1B/1D, 5-HT1F) [◊]	- cognitive impairment at complex reaction time tasks, cognitive flexibility tests, and verbal memory in ergotamine abusers.	[19,20,21,231,232,233,234,235]

Abbreviations: Aβ, β-amyloid peptide; AD, Alzheimer’s disease; Akt/GSK-3β/CREB, protein kinase B/glycogen synthase kinase-3β/cAMP response element binding protein; ApoE, apolipoprotein E; APP, amyloid precursor protein; ARB, angiotensin (AT) II receptor blocker; ASMs, anti-seizure medications; BIBN, CGRP receptor antagonist; BDNF, brain-derived neurotrophic factor; cAMP, cyclic adenosine monophosphate; CGRP, calcitonin-gene-related peptide; DHE, dihydroergotamine; GBP, gabapentin; IL-1β, interleukin 1 beta; Kd, dissociation constant; MCI, mild cognitive impairment; MMP-2, matrix metallopeptidase 2; MMSE, Mini-Mental State Examination; PI3K/Akt/mTOR, phosphatidylinositol 3-kinase/protein kinase b/mammalian target of rapamycin; TGF-β1, transforming growth factor beta 1; TNF-α, tumor necrosis factor alpha; TPM, topiramate; TrkA and TrkB, tyrosine protein kinase (neurotrophin) receptors A and B; VPA, valproic acid; 5-HT HT1B/1D/1F, serotonin receptor subtypes. * cognitive effects tested on animal models. Investigations on samples from: [§] rats, [#] mice, [†] or other species/media. Clinical studies are indicated by the symbol [◊].

## Supplementary Materials

The following supporting information can be downloaded at https://www.mdpi.com/article/10.3390/ijms231911418/s1.
