# Supplementary material for: Migraine Pharmacological Treatment and Cognitive Impairment: Risks and Benefits
Source: Int J Mol Sci. 2022 Sep 27;23(19):11418. doi: 10.3390/ijms231911418 (PMC9569564; doi:10.3390/ijms231911418)
Supplement: Supplementary file 1 [file ijms-23-11418-s001.zip › ijms-1881971-supplementary-final.pdf]

Supplementary Table S1. Clinical trials on anti-migraine compounds.

Study	IP	Study Design	Sample	Main findings
Kockelmann E et al. 2004[45]	TPM<br>LTG	Retrospective comparative study	TPM= 42 PwE<br>LTG= 42 PwE	Polytherapies containing TPM were associated with worse executive functions.
Kim SY et al. 2006[46]	TPM<br>OXC	Retrospective comparative study	TPM= 30 PwE<br>OXC= 30 PwE	Patients receiving low-dose TPM for 1 year showed worse working memory and verbal fluency.
Fritz N et al. 2005[47]	TPM<br>TGB	Open prospective randomized study	TPM= 15 PwE<br>TGB= 15 PwE	TPM add-on was associated with worsening of language capacities (fluency and comprehension) and short-term visuospatial working memory.
Fröscher W et al. 2005[48]	TPM	Prospective study	42 PwE	Patients who experienced abnormal thinking, impaired concentration, speech problems, or somnolence were under higher TPM dosages and showed higher serum concentrations than those who did not report these AEs.
Gomer B et al. 2007[49]	TPM<br>LTG	Open prospective randomized study	TPM= 21 PwE<br>LEV= 30 PwE	Decreased spatial and verbal short-term memory, cognitive speed, and verbal fluency were observed in TPM-treated patients.
Jung KY et al. 2010[50]	TPM	Prospective study	18 PwE	After starting TPM, the patients' performances declined in terms of short-term visuospatial and verbal memory, working memory, and verbal fluency (phonemic and semantic).
Naegel S et al. 2009[51]	TPM	Prospective study	29 PwE with mental retardation	A decrease in concentration was observed in 38% of subjects. Alertness worsened in 24% of cases.
Kanner AC et al. 2003[54]	TPM	Prospective study	596 PwE	CAEs were found in 41% of treated subjects, with a significant association with a past psychiatric history of depression.
Wandschneider B et al. 2017[55]	TPM<br>ZNS<br>LEV	Retrospective, cross-sectional study	TPM= 21 PwE<br>ZNS= 51 PwE<br>LEV= 62 PwE	Worse cognitive performance in TPM-treated subjects.
Meador KJ et al. 2003[57]	TPM<br>VPA	Multicenter, randomized, double-blind study	TPM= 34 PwE<br>VPA= 29 PwE<br>PBO= 13 PwE	After three months of TPM treatment, a lower performance was observed at the SDMT and COWAT tests, indicating a deterioration of processing speed and verbal fluency.
Prevey ML et al. 1996[58]	CBZ<br>VPA	Prospective randomized double-blind multicenter study	CBZ= 26 PwE<br>VPA= 39 PwE<br>HC= 72	No cognitive worsening was observed in the two treated arms. However, a lack of learning/practice effect, compared to HC, was noted.
Ristić AJ et al. 2006[59]	VPA	Open prospective study	364 PwE	The incidence of parkinsonism and CAEs was very low (1.37% and 0.62%, respectively). CAEs included memory deficits and psychomotor slowness.
Dodrill CB et al. 1999[63]	GBP	Multicenter, randomized, double-blind, parallel-group, dose-controlled study	GBP= 201 PwE<br>PBO= 85 PwE	No cognitive effects were observed.

Meador KJ et al. 1999[64]	GBP<br>CBZ	Double-blind, randomized crossover study	35 HC	GBP treatment is associated with fewer CAEs than CBZ.
Leach JP et al. 1997[65]	GBP	Double-blind, randomized, dose-ranging, placebo-controlled, crossover study	27 PwE	GBP showed no significant effect on cognition. Only drowsiness was increased at the highest dosage.
Salinsky MC et al. 2002[66]	GBP<br>CBZ	Double-blind, randomized, parallel-group study	GBP= 12 HC<br>CBZ= 11 HC<br>CTRL= 72 HC	AED treatment was associated with worse subjective cognitive performances. Objective measures only showed slight changes upon AEDs, with a small effect size.
Shem K et al. 2018[67]	GBP	Prospective observational cohort study	10 PwSI	Decreased attention, memory, executive functions, and verbal fluency were observed, along with increased working memory.
Salinsky MC et al. 2010[71]	PGB	Double-blind, parallel-group, randomized, placebo-controlled study	PGB= 16 HC<br>PBO= 16 HC	PGB-treat subjects showed mild worsening of attention, compared to controls.
Placidi F et al. 2000[74]	LTG	Open prospective study	13 PwE	LTG did not increase daytime somnolence and improved sleep quality.
Ciesielski AS et al 2006[77]	PGB<br>LEV	Open prospective comparative study	PGB= 13 PwE<br>LEV= 13 PwE	PGB-treated patients showed mild worsening of visual short-term memory. Instead, LEV add-on did not cause cognitive changes. In the direct comparison between LEV-treated and PGB-treated patients, non-significant trends indicated better cognitive profiles in the first group.
López-Góngora M et al. 2008[78]	LEV	Open prospective study	27 PwE	Improvement in several cognitive domains was observed (attention, prospective memory, working memory, and verbal fluency).
Park SP et al. 2007[80]	ZNS	Retrospective cross-sectional study	60 PwE	Subjective cognitive deficits were frequently reported (e.g., 35% memory, 27% attention).
Pfistermeister B et al. 2017[87]	AMT	Retrospective cross-sectional study	Total= 89 579<br>AMT= 1684	AMT is commonly prescribed to the elderly, despite the high Anticholinergic Cognitive Burden (listed as class 3).
Ibor JJ et al. 2008[94]	VXR	Prospective, multicenter, naturalistic, open-label study	59 PwD	Improvement of global cognitive functioning was observed upon VXR therapy.
Hedayat M et al. 2022[96]	VLF<br>AMT	Noninferiority randomized double-blind study	VLF= 40 PwM<br>AMT= 40 PwM	While 37.5% of AMT-treated patients reported increased sleepiness, only 15% of VLF-patients complained of the onset of confusion.

Shilyansky C et al. 2016[98]	ESC<br>SER<br>VXR	Multicenter, open-label, randomized, prospective study	ESC = 336 PwD<br>SER = 336 PwD<br>VXR= 336 PwD<br>336 HC	Only responders showed some improvement in terms of visuospatial planning and cognitive flexibility. Non-responders did not benefit from any antidepressant treatment. The three drugs had similar effects.
Greer TL et al. 2014[101]	DLX	Open prospective study	30 PwD	DLX-treated patients showed better performances in several cognitive domains. The cognitive enhancement was independent of the antidepressant effect in all cases, except for verbal learning and memory.
Mowla A et al. 2007[108]	FXT	Double-blind, placebo-controlled study	FXT= 29 MCI<br>PLB= 29 MCI	FXT treatment improved global cognition and verbal memory for prose.
Holm et al. 2020[114]	BBs	Prospective population-based study	BBs= 3720<br>Non-BBs= 3720	BB users had a higher risk of developing VaD, but not AD or MixD.
Gliebus G and Lippa CF. 2007[116]	BBs	Retrospective cross-sectional study	Total= 64 PwCI<br>(CNS-BBs= 20 PwCI)	A trend of lower global cognitive scores and memory retrieval was observed in patients under therapy with CNS-BBs.
Beaman EE et al. 2022[117]	BBs	Retrospective cross-sectional study	Total= 69 081	The use of CNS-BBs was associated with a lower risk of developing AD, compared to L-CNS-BBs.
Müller U et al. 2005[121]	PRO<br>ATE	Double-blind, balanced placebo-controlled study	24 HC	PRO administration impaired cognitive performances at a working memory task by slowing the reaction times.
Palac DM et al. 1990[122]	PRO<br>ATE	Double-blind, randomized controlled crossover	42 HT	Neither drug significantly affected the subjects' cognition.
Madden DJ et al. 1988[123]	PRO<br>ATE	Open prospective study	PRO= 9 HT<br>ATE= 7 HT<br>PBO= 8 HT	No significant difference, in short-term memory, was found in treated patients compared to PBO.
Pérez-Stable EJ et al. 2000[124]	PRO	Double-blind, randomized, placebo-controlled study	PRO= 152 HT<br>PBO= 152 HT	PRO administration caused a mild long-term impairment of sustained attention.
Wikstrand J et al. 1988[127]	MET	Prospective, randomized study	MET= 1609 HT<br>THZ= 1625 HT	The risk of cardiovascular death was lower in MET-treated patients than in those receiving thiazides.
Streufert S et al. 1988[128]	MET<br>ATE	Double-blind, randomized crossover study	MET= 25 HT<br>ATE= 25 HT	MET-treated patients showed better performances in proofreading, visuomotor tasks, and executive functions. ATE-treated patients' performances were comparable to or slightly worse than PBO.
Fogari R et al. 2003[132]	ATE<br>LOS	Double-blind, randomized, parallel design	ATE= 60 HT<br>LOS= 60 HT	LOS administration improved verbal memory performance compared to baseline and ATE-treated patients. ATE administration did not induce any significant change in test scores.
Muldoon MF et al. 2002[134]	ATE<br>MET<br>VER<br>Others	Double-blind, randomized, prospective study	98 HT<br>32 HC	All anti-HT treatments improved the working memory-related tasks, but impaired motor speed, cognitive flexibility, and perceptual motor velocity.

Lin W et al. 2019[138]	FZ	Nationwide population-based Study	6 470 PwM	FZ exposure increases the risk of parkinsonism.
Parrott AC and Wesnes K 1987[153]	CZ	Double-blind, placebo-controlled study	12 HC	CZ administration impaired psychomotor performance with a maximal effect after 5-6 hours.
Gordon CR et al. 2001[154]	CZ	Double-blind, placebo-controlled, randomized, crossover study	60 HC	CZ did not affect cognitive performances.
Ashrafi MR et al. 2014[155]	CZ	Double-blind, placebo-controlled, parallel-group randomized study	CZ= 34 PwM<br>PBO= 34 PwM	No CAEs or iatrogenic parkinsonism were noted. 8.82% of CZ-treated PwM reported mild drowsiness, compared to 2.95% of PBO.
Ciancarelli et al. 2004[158]	FZ	Prospective comparative study	25 PwM<br>25 HC	FZ does not interfere with NO-related vasodilatation.
Wharton W et al.2015[169]	RAS	Open retrospective study	RAS= 488 MCI<br>Non-RAS= 296 MCI	The use of RAS agents, especially those with BBB penetration, was associated with lower rates of conversion to AD and better cognitive profiles. The effect was more prominent in African Americans than in Caucasians.
Barthold D et al. 2018[170]	RAS	Retrospective comparative study	Total= 1 343 334 HT<br>RAS= 583 182 HT	RAS-treated male patients had a lower risk of developing AD than those receiving other anti-hypertensive medications.
Lithell H et al. 2003[175]	CAN	Prospective, double-blind, randomized, parallel-group study	CAN= 2477 HT<br>Controls= 2460 HT	CAN administration did not significantly affect cognition.
Saxby et al. 2008[176]	CAN	Double-blind, placebo-controlled, randomized study	CAN= 128 HT<br>PBO= 129 HT	Patients receiving CAN showed an inferior rate of decline in attention and episodic memory.
Bosch J et al. 2019[177]	CAN	Double-blind, randomized, placebo-controlled	CAN/HCT/ROS=587 HC<br>ROS/PBO= 594 HC<br>CAN/HCT/PBO= 593 HC<br>PBO/PBO= 587 HC	CAN administration, either with HCT alone or HCT+ROS, did not significantly affect the patients' cognition.
Hajjar I et al. 2013[178]	CAN	Double-blind randomized study	CAN= 17 HT<br>LIS= 17 HT<br>HCT= 17 HT	CAN-treated patients showed greater improvement in executive tasks compared to the other two arms.
Hajjar I et al. 2020[179]	CAN	Double-blind, randomized study	CAN= 87 MCI<br>LIS= 89 MCI	CAN-treated patients showed better cognitive performances (verbal memory and some executive functions) than those receiving LIS.
Deen M et al. 2019[207]	SUM	Prospective open study	8 PwM	SUM administration reduced 5-HT_{1B} receptor binding in brain regions linked to pain modulation.

Farmer K et al. 2001[227]	SUM	Open-label, single-attack study	28 PwM	SUM administration relieved the transitory cognitive impairment associated with migraine attacks.
Evers S et al. 2003[232]	SUM<br>ZOL<br>ERG	Double-blind, placebo-controlled, crossover study	16 HC	ERG administration significantly increased N2 latency at visually evoked event-related potentials. Mild worsening at cognitive tasks was observed 2 hours after the intake of ERG and – to a lesser degree – ZOL.
Roon KI et al. 2000[233]	ERG	Prospective study	ERG abusers= 12 PwM<br>12 HC	ERG abusers showed poorer processing speed and cognitive flexibility.
Chang CW et al. 2016[239]	ASA	Nationwide, population-based retrospective study	ASA= 2 876 T2DM<br>Non-ASA= 10 720 T2DM	T2DM patients who were receiving low-dose ASA therapy showed a lower risk of developing AD.
Côté S et al. 2012[240]	NSAIDs	Nationwide, population-based, prospective study	Total= 5276	Patients receiving NSAID therapy were at lower risk of all-cause dementia, AD, and non-dementia cognitive impairment.
Wichmann MA et al. 2016[245]	ASA<br>Other NSAIDs	Population-based, prospective study	Total= 5924	ASA intake was associated with an increased long-term risk of developing dementia, whereas non-ASA NSAID therapy did not show any significant association with the risk of cognitive impairment.
Ancelin ML et al. 2012[246]	NSAIDs	Multi-center, prospective, cohort study	Total= 7 486	No significant association between NSAID therapy and cognitive impairment was found.
Breitner JCS et al. 2009[247]	NSAIDs	Prospective, community-based study	Total= 2 736	Increased incidence of AD was observed among heavy users of NSAIDs.
Roumie CL et al. 2008[249]	NSAIDs	Retrospective cohort study	Total= 336 906	Treatment with rofecoxib or valdecoxib increased the risk of stroke.

AD= Alzheimer's Disease (patients with); AED= Anti-epileptic drug; AMT= Amitriptyline; ASA= Acetyl Salicylic Acid; ATE= Atenolol; BBs= Beta-blockers; CAEs= Cognitive Adverse Effects; CAN= Candesartan; CBZ= Carbamazepine; CNS-BBs= beta-blockers with high blood-brain barrier penetration like propranolol, metoprolol, or carvedilol; COWAT= Control Word Association; CZ= Cinnarizine; DLX= Duloxetine; ERG= Ergotamine; ESC= Escitalopram; FLX= Fluoxetine; FZ= Flunarizine; GBP= Gabapentin; HC= Healthy Controls; HCT= Hydrochlorothiazide; HT= Hypertension (patients with); L-CNS-BBs= beta-blockers with low blood-brain barrier penetration like bisoprolol, atenolol, or sotalol; LEV= Levetiracetam; LIS= Lisinopril; LOS= Losartan; LTG= Lamotrigine; MCI= Mild Cognitive Impairment (patients with); MET= Metoprolol; MixD= Mixed Dementia; NSAIDs= Non-Steroidal Anti-Inflammatory Drugs; OXC= Oxcarbazepine; PBO= Placebo; PGB= Pregabalin; PRO= Propranolol; PwCI= Patients with Cognitive Impairment; PwD= Patients with Dementia; PwE= Patients with Epilepsy; PwSI= Patients with Spinal Injury; RAS= Drugs acting on the Renin-Angiotensin System; ROS= Rosuvastatin; SDMT= Symbol Digit Modalities Test; SER= Sertraline; SUM= Sumatriptan; TGB= Tiagabine; THZ= Thiazides; TPM= Topiramate; VER= Verapamil; VLF= Venlafaxine; VPA= Valproic Acid; VXR= Venlafaxine Extended Release; ZNS= Zonisamide; ZOL= Zolmitriptan.
